# The impact of light during the night

**DOI:** 10.7554/eLife.52364

**Published:** 2019-11-12

**Authors:** Sophia TC Leung, R Anne McKinney, Alanna J Watt

**Affiliations:** 1 Department of BiologyMcGill UniversityMontrealCanada; 2 Department of Pharmacology and TherapeuticsMcGill UniversityMontrealCanada

**Keywords:** steroid, brain development, pineal gland, light-at-night, Chicken

## Abstract

Exposing chicks to one hour of light during the night disrupts the release of a hormone that is needed by cells in the developing brain to survive.

**Related research article** Haraguchi S, Kamata M, Tokita T, Tashiro KI, Sato M, Nozaki M, Okamoto-Katsuyama M, Shimizu I, Han G, Chowdhury VS, Lei XF, Miyazaki T, Kim-Kaneyama JR, Nakamachi T, Matsuda K, Ohtaki H, Tokumoto T, Tachibana T, Miyazaki A, Tsutsui K. 2019. Light-at-night exposure affects brain development through pineal allopregnanolone-dependent mechanisms. *eLife* **8**:e45306. doi: 10.7554/eLife.45306

Modern life exposes us to multiple sources of artificial light at times when we normally would experience darkness. You could, for example, be reading this article at night on the glowing screen of a computer or tablet. There is mounting evidence, however, that irregular 'light-at-night' can affect mood and brain function (Bedrosian and Nelson, 2017). The developing brain is particularly sensitive to night-time light (Logan and McClung, 2019), and this could result in changes that persist into adult life.

Now, in eLife, Shogo Haraguchi, Kazuyoshi Tsutsui and colleagues at Waseda University, Showa University School of Medicine and other institutes in Japan report that inappropriate light-at-night can have adverse long-term effects on the survival of nerve cells in the developing brain of chicks (Haraguchi et al., 2019). Chicks are an ideal model system for studying how light affects the developing brain because, like people, chickens are diurnal (i.e., they are active during the day and sleep at night).

Haraguchi et al. focused on a small gland located deep in the brain called the pineal gland, which regulates the sleep-wake cycle by producing and releasing a hormone known as melatonin (Borjigin et al., 2012). The pineal gland synthesizes melatonin in rhythmic cycles, releasing low levels during the day and high levels at night. However, the release of melatonin during the night can be suppressed by exposure to bright light. In addition to melatonin, the pineal gland also produces and releases various steroids, including a steroid called ALLO (short for allopregnanolone; Haraguchi et al., 2012).

Haraguchi et al. showed that when hatchling chicks were repeatedly exposed to 12 hours of light followed by 12 hours of darkness, ALLO expression in the pineal gland peaked four hours into the dark cycle (at hour 16 of the 24-hour cycle). However, when the chicks were exposed to constant light, ALLO levels remained low and the peak in expression during the dark cycle could no longer be detected. Notably, exposing chicks to just one hour of light during the night (14-15 hours into the cycle) was enough to abolish the peak in ALLO synthesis (Figure 1).

**Figure 1.**
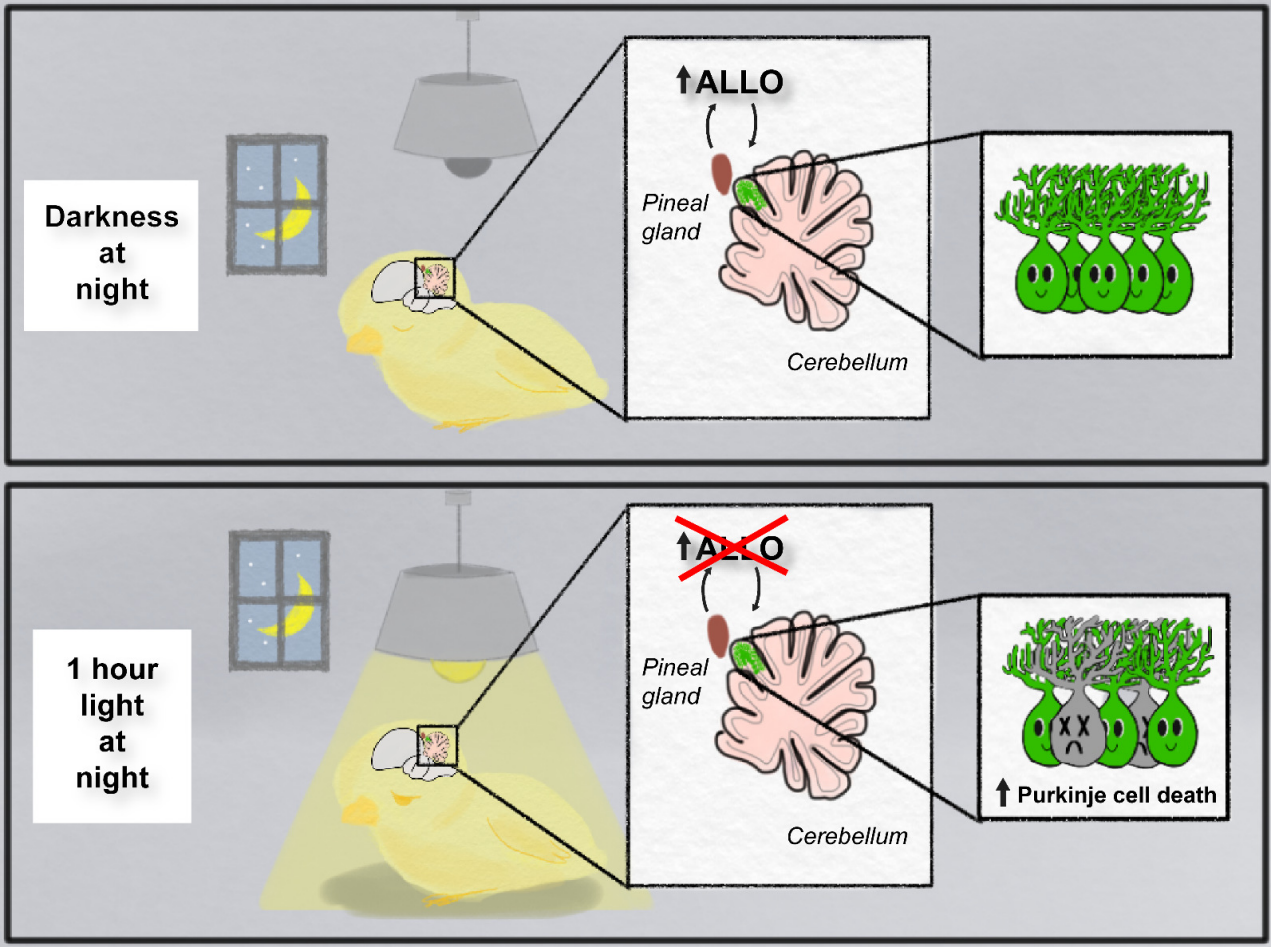
Light-at-night and the developing brain. When developing chicks are exposed to repeated cycles of 12 hours of light and 12 hours of darkness (top panel), they release a hormone called ALLO from the pineal gland during the dark phase. However, when the chicks were exposed to one hour of light during the night for a week (bottom panel), the release of ALLO was disrupted, and this resulted in the death of Purkinje cells in the part of the cerebellum that is next to the pineal gland (live cells are shown in green; dead cells are shown in gray).

Image credit: Sophia Leung

Next, Haraguchi et al. determined how changes in ALLO expression affected the development of Purkinje cells in the cerebellum: this region of the brain, which is located next to the pineal gland, is important for motor coordination and cognition (Koziol et al., 2014). They found that exposing chicks to constant light, or one hour of aberrant light per night, the week after hatching caused an increase in the death of Purkinje cells: moreover, these losses were observed predominantly in the part of the cerebellum closest to the pineal gland and persisted into young adulthood (Figure 1). Further experiments showed that the Purkinje cells did not die when chicks exposed to one hour of light per night were injected with a daily dose of ALLO. This suggests that Purkinje cells in the developing brain need ALLO in order to survive.

To determine the underlying mechanisms that cause Purkinje cell death, Haraguchi et al. carried out a series of experiments in which they down-regulated the expression of specific proteins in the egg. This revealed that ALLO acted via a receptor on the cell membrane known as mPRα, and this led to daily peaks in expression of a neuroprotective hormone called PACAP during the dark phase of the cycle. Epigenetic regulation of the gene that controls PACAP during development caused expression levels of this hormone to vary between chicks. Chicks exposed to night-time light in the second week after hatching also experienced Purkinje cell death. However, the damage caused by this later exposure could be rescued by PACAP, but not by the addition of ALLO. This suggests that the actions of light-at-night in the brain may involve different pathways at different stages of development.

Purkinje cell death is observed in several neurological disorders, including autism (Jayabal and Watt, 2019), and the pattern of Purkinje cell death following light-at-night exposure during development is similar to the patterns of cell death observed in several ataxia disorders that affect motor coordination (Langmade et al., 2006; Larivière et al., 2015). Since disrupted sleep is a common component of many ataxia disorders (Huebra et al., 2019), this study suggests that cell survival in neurogenerative diseases may be linked to changes in neurosteroid production caused by interrupted sleep.

Caution, however, must be taken when translating these results to humans, since the stages of brain development in chicks are not directly comparable to humans. Nonetheless, this study raises important concerns about how light exposure at night affects the developing brain. In addition to helping you get a good night’s sleep, turning off screens and shutting blinds at night may help maintain the production of a neurosteroid that cells in the developing brain need to survive.
